# When to perform curettage after uterine artery embolization for cesarean scar pregnancy: a clinical study

**DOI:** 10.1186/s12884-021-03846-x

**Published:** 2021-05-10

**Authors:** Qiao Wang, Hongling Peng, Xia Zhao, Xiaorong Qi

**Affiliations:** Department of Gynecology and Obstetrics, Development and Related Diseases of Women and Children Key Laboratory of Sichuan Province, Key Laboratory of Birth Defects and Related Diseases of Women and Children, Ministry of Education, West China Second Hospital, Sichuan University, Chengdu, Sichuan 610041 People’s Republic of China

**Keywords:** Cesarean scar pregnancy, Uterine artery embolization, Curettage, Treatment interval, Hemorrhage

## Abstract

**Background:**

Prophylactic uterine artery embolization (UAE) combined with subsequent curettage is suggested as an effective and minimally invasive treatment strategy for cesarean scar pregnancy (CSP) with a high bleeding risk. However, the timing of curettage after UAE remains to be studied. Thus, we aimed to identify the optimal time interval to perform curettage after UAE in patients with CSP.

**Methods:**

We conducted a retrospective cohort study in a large medical center for women and children in Southwest China. CSP patients treated by UAE combined with subsequent curettage were included and grouped by the treatment time interval between these two procedures. The clinical outcomes among arms were compared by univariate and multivariable analysis.

**Results:**

Our study included 314 CSP patients who received this combination treatment in our department from January 2014 to December 2019. The median time interval between UAE and curettage was 48 h, with a range of 12-168 h among all participants. Thirty-two patients (10.2%) experienced intraoperative hemorrhage (blood loss ≥200 mL). Intrauterine balloon tamponade was used in 17 cases (5.4%). In 14 cases (4.5%), the procedure was converted to laparoscopy (or laparotomy). In the cohort study, patients with longer treatment intervals had more intraoperative blood loss and a higher incidence of complications than those with shorter intervals (*P* < 0.05). The rates of intraoperative bleeding were 5.0% for patients who received curettage within 24 h after UAE (Arm 1) and 19.4% for those who had a treatment interval longer than 72 h (Arm 4). In the multivariable logistic regression model of bleeding, a treatment interval > 72 h had an adjusted odds ratio of 3.37 (95% confidence interval: 1.40-8.09).

**Conclusion:**

We suggest that curettage not be delayed longer than 72 h after UAE in this combined treatment of CSP.

## Background

Cesarean scar pregnancy (CSP) is a late complication of cesarean section (CS) and is defined as an early pregnancy implanting in the scar from the prior CS [1]. CSP is considered a rare type of ectopic pregnancy, but its incidence is increasing due to increases in the CS rate, especially in developing countries [2]. Since the gestational sac is implanted in the scar tissue, which is a weak area in the lower segment of the uterus, CSP can cause life-threatening conditions such as uncontrollable bleeding and uterine rupture, and increase maternal mortality [3].

Currently, there is no optimal treatment recommendation for CSP [1]. The management of CSP should be individualized [4]. According to previous studies, adjuvant uterine artery embolization (UAE) combined with subsequent curettage (including dilatation and curettage or vacuum aspiration) was suggested as an effective and minimally invasive treatment strategy for CSP patients with high bleeding risk [5–7]. Prophylactic UAE can reduce the risk of severe bleeding during curettage by pretreatment with local methotrexate (MTX) administration and blockage of the main blood supply. The efficacy and safety of UAE have been evaluated by numerous studies. However, the timing of curettage after UAE remains to be studied. In previously reported studies, the time intervals between UAE and curettage were quite different, ranging from 24 to 72 h [5–11]. There is no consensus on the optimal time interval to perform curettage after UAE.

We therefore conducted the present clinical study to investigate the relationships between clinical outcomes and the treatment interval between UAE and curettage in CSP patients, aiming to identify the most appropriate timing to perform curettage after UAE.

## Methods

### Subjects

This study was approved by the Institutional Review Board of Ethics Committee of West China Second Hospital, Sichuan University, People’s Republic of China. West China Second Hospital is one of the largest gynecology and obstetrics medical centers in Southwest China. We recruited all patients diagnosed with CSP who received the combination treatment of adjuvant UAE and ultrasound-guided curettage in our hospital from January 2014 to December 2019. Two branch courts participated in this study. Signed informed consent forms were obtained from each patient whose clinical data were collected.

### Diagnosis

CSP was diagnosed depending on (1) a history of transverse lower segment cesarean section; (2) a positive blood test of serum beta-human chorionic gonadotrophin (beta-hCG); and (3) ultrasound imaging. A ultrasonographic diagnosis was made based on the following criteria [12]: (1) empty uterine cavity and cervical canal; (2) a gestational sac, with or without fetal cardiac activity, located in the anterior portion of the lower uterine segment (the scar of a prior CS); and (3) a thin (≤3 mm) or absent myometrium between the gestational sac and the bladder. We classified the CSP cases into two types depending on ultrasound imaging and magnetic resonance imaging (MRI): (1) type I (endogenic type) with progression toward the uterine cavity and (2) type II (exogenic type) with progression toward the bladder [13]. MRI was performed to assess a cesarean scar defect and identify the trophoblastic layer and the myometrium separately, to guide the decision-making process for the treatment strategy. Thus, pelvic MRI was performed for every CSP patient who needed prophylactic UAE treatment, as required by radiologists.

### Inclusion and exclusion criteria

Our study included CSP patients who met the following criteria: (1) gestation of 5–12 weeks; (2) diagnosis and clarification of CSP confirmed by both transvaginal Doppler ultrasound and MRI [8, 14]; (3) UAE within 48 h after diagnosis; and (4) ultrasound-guided curettage following pretreatment with UAE. We excluded patients (1) who had ever received failed surgical or medical treatment before transfer to our department or (2) who had maternal hepatic, renal, or blood system diseases. The type of gelfoam (with diameters of 1–3.0 mm, Alicon, Hangzhou, China) and dose of MTX (50 mg/m^2^ body surface area) used in UAE were consistent in each case. MTX was bilaterally injected through the uterine arteries followed by the main stems of uterine arteries blocked by gelfoam particles. All UAE and curettage procedures were performed by specialists in our hospital following the same protocol.

### Study design

This was a retrospective study designed as a cohort study to evaluate clinical outcomes among four arms that were grouped by the time interval between UAE and curettage. Medical records and clinical data of every patient meeting the inclusion criteria were reviewed. Characteristics extracted as variables in the analysis included maternal age, gravidity, parity, number of prior CSs, time interval between last-time CS and present CSP, presence of fetal cardiac activity, level of serum beta-hCG, gestational age, diameters of gestational sac (or CSP mass), thickness of myometrium between the gestational sac and the bladder, ultrasonographic presence of peritrophoblastic blood supply, and the type of CSP. In addition, we took the recurrence of CSP as one of the variables in the analysis. The primary outcome in our study was intraoperative blood loss. The bleeding volume was measured by weighing the blood captured in the suction device and under-buttocks pad. Other clinical outcomes included the incidence of complications and the total length of hospital stay. Clinical events recorded as complications were (1) usage of intrauterine balloon tamponade to control active bleeding during or after curettage; (2) excessive hemorrhage with blood transfusion; (3) persistent CSP; (4) subsequent laparoscopy or laparotomy due to uterine rupture or uncontrollable bleeding; and (5) surgical infection. Statistical reviews between the bleeding group and control group were further conducted to identify risk factors for intraoperative bleeding. The bleeding group included cases with blood loss ≥200 mL during curettage. Patients with intraoperative blood loss less than 200 mL were included in the control group.

### Statistical analysis

The data analysis was conducted by IBM SPSS Statistics 23.0 software. Data were appropriately analyzed by parametrical tests or nonparametrical tests. Continuous variables are reported as the mean ± standard deviation (SD). Discrete variables are reported as medians (interquartile ranges, IQRs). Data with a normal distribution were compared by independent-sample *t* tests between two groups. Data with a nonnormal distribution were compared by means of rank sum tests between two groups. Categorical variables are reported as numbers (%) and were compared by means of chi-square (*x*^*2*^*)* tests between two groups. Comparisons of data among multiple arms were tested by analysis of variance (ANOVA). A *P* value < 0.05 was considered statistically significant. Confounding factors were adjusted in the multivariable analysis. A multivariable binary logistic regression model was conducted to evaluate the relationship between variables and intraoperative bleeding. Odds ratios (ORs) and 95% confidence intervals (CIs) of each variable were calculated to identify the risk factors for bleeding (if both the OR and 95% CI were > 1).

## Results

**Fig. 1:**
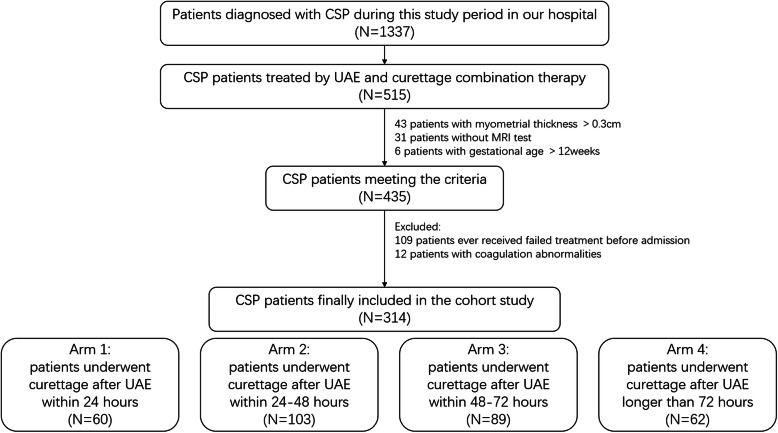
Flow chart of participants. Our study included 314 CSP patients who were treated with the combination of UAE and curettage. We divided the participants into four arms depending on the time interval between UAE and curettage. CSP = cesarean scar pregnancy. UAE = uterine artery embolization. MRI = magnetic resonance imaging

Our study finally included a total of 314 CSP patients who were treated with a combination of UAE and curettage between January 2014 and December 2019 (Fig. 1). Among them, 46 cases (14.6%) had the following regimens for at least one complication: use of an intrauterine balloon in 17 cases (5.4%) with active bleeding without uterine rupture and conversion to emergency laparoscopy or laparotomy for uterine repair in 14 cases (4.5%) with active bleeding and uterine rupture. We found persistent CSP in 8 cases (2.5%), who received subsequent treatment as systemic MTX or a second sugery. Five cases (1.6%) required blood transfusion. Only one patient (0.3%) underwent hysterectomy because of uncontrollable bleeding.

**Fig. 2:**
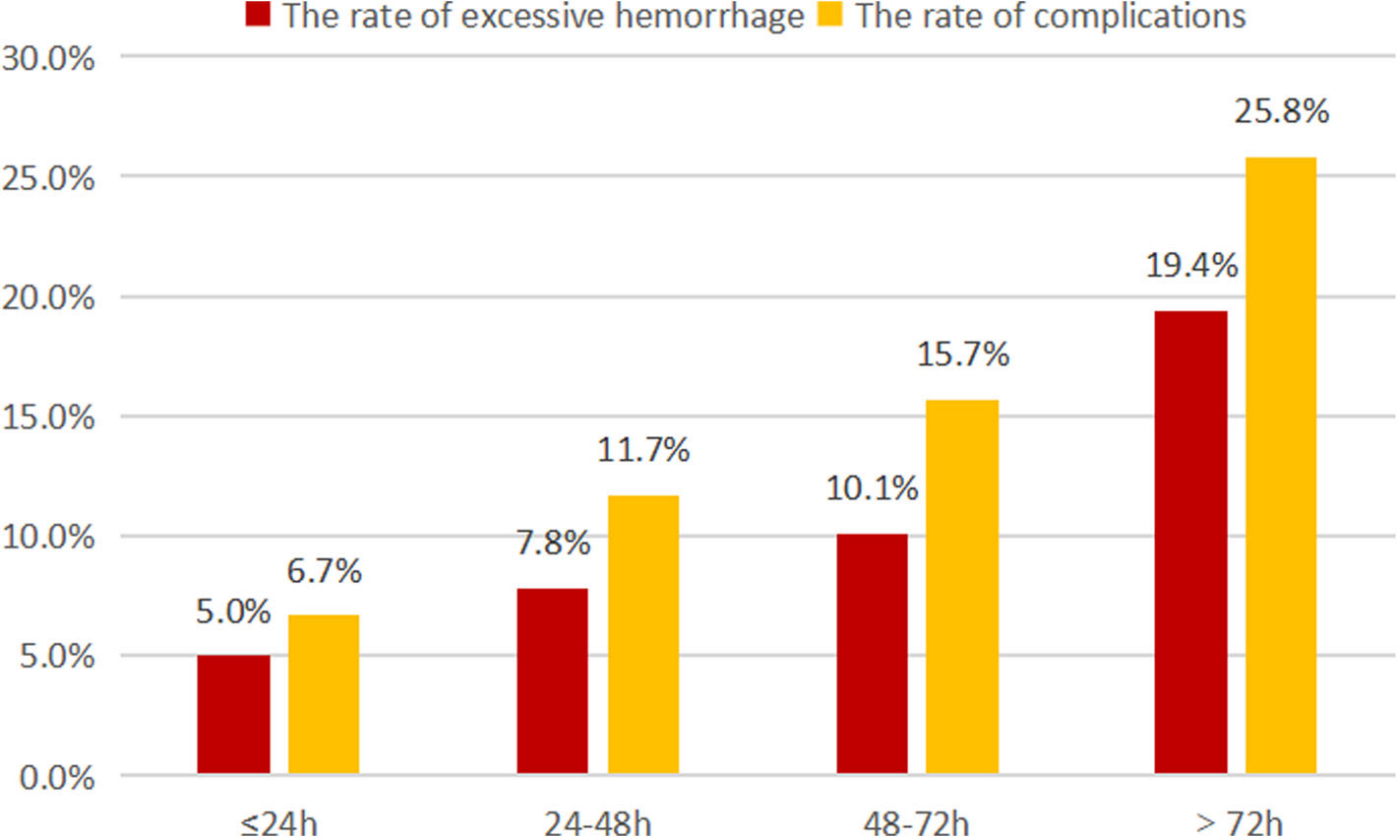
Differences in clinical outcomes of each arm. CSP patients treated with a longer time interval between UAE and curettage were likely to have a higher rate of excessive hemorrhage and other complications. CSP = cesarean scar pregnancy. UAE = uterine artery embolization

Among all participants, the median treatment interval between the two procedures was 48 h, with a range of 12-168 h. In the cohort analysis, patients were divided into four arms depending on the time interval between UAE and curettage. The baseline patient characteristics and clinical outcomes of each arm are listed in Table 1. There were no significant differences in the baseline characteristics of patients among the different arms (*P* > 0.05). However, we found that CSP patients who had longer treatment intervals were likely to have worse clinical outcomes (more intraoperative blood loss, a higher rate of complications, and longer hospital stays (*P* < 0.05) than those in the shorter interval arms (Fig. 2). The rates of excessive hemorrhage were 5.0% among patients who received curettage within 24 h after UAE (Arm 1) and 19.4% among those who had a treatment interval longer than 72 h (Arm 4).

**Table 1:** Cohort study: baseline patient characteristics and clinical outcomes in each arm

Variable	Total	Interval between UAE and curettage				***P*** value
		Arm 1	Arm 2	Arm 3	Arm 4	
		≤24 h	24-48 h	48-72 h	> 72 h	
	***N*** = 314	***N*** = 60	***N*** = 103	***N*** = 89	***N*** = 62	
**Maternal age (years)**^**a**^	32.4 ± 3.8	33.3 ± 4.3	31.8 ± 4.8	32.8 ± 4.9	31.4 ± 5.3	0.074
**Gravidity**^**b**^	4 (1-12)	5 (2-11)	4 (1-11)	4 (2-9)	4 (1-12)	0.870
**Parity**^**b**^	1 (1-3)	1 (1-3)	1 (1-3)	1 (1-3)	1 (1-3)	0.978
**Number of prior CS**^**b**^	1 (1-3)	1 (1-3)	1 (1-3)	1 (1-3)	1 (1-2)	0.981
**Interval between CS and CSP (years)**^**a**^	5.8 ± 3.9	6.4 ± 3.9	5.4 ± 3.9	6.0 ± 4.0	5.2 ± 3.8	0.209
**Recurrent CSP (number, %)**^**c**^	11 (3.5%)	5 (8.3%)	3 (2.9%)	1 (1.1%)	2 (3.2%)	0.125
**Fetal heart beat (number, %)**^**c**^	112 (35.7%)	26 (43.3%)	38 (36.9%)	29 (32.6%)	19 (30.6)	0.450
β-hCG level (*10^5mIU/mL)^**a**^	5.79 ± 6.93	6.40 ± 4.76	4.76 ± 4.47	6.08 ± 9.33	6.52 ± 8.04	0.323
**Gestational age (days)**^**a**^	51.3 ± 10.9	50.0 ± 8.8	51 ± 10.9	52.1 ± 12.2	52.0 ± 11.2	0.645
**Maximum diameter of CSP mass (cm)**^**a**^	3.2 ± 1.6	3.2 ± 1.7	3.1 ± 1.6	3.3 ± 1.6	3.0 ± 1.4	0.624
**Median diameter of CSP mass (cm)**^**a**^	2.4 ± 1.3	2.4 ± 1.2	2.5 ± 1.3	2.6 ± 1.4	2.4 ± 1.3	0.797
**Minimum diameter of CSP mass (cm)**^**a**^	1.8 ± 1.2	1.7 ± 1.1	1.9 ± 1.1	2.0 ± 1.3	1.9 ± 1.2	0.730
**Myometrial thickness (cm)**^**a**^	0.11 ± 0.08	0.11 ± 0.09	0.11 ± 0.09	0.11 ± 0.08	0.11 ± 0.08	0.978
**Rich blood supply (number, %)**^**c**^	56 (17.8%)	10 (16.7%)	24 (23.3%)	15 (16.9%)	7 (11.3%)	0.131
**Type of CSP**						
Type I (number, %)^c^	263 (83.8%)	54 (90%)	85 (82.5%)	70 (78.7%)	54 (87.1%)	0.252
Type II (number, %)^c^	51 (16.2%)	6 (10%)	18 (17.5%)	19 (21.3%)	8 (12.9%)	
**Clinical outcome**						
**Intraoperative blood loss (mL)**^**a**^	66.7 ± 160.1	44.5 ± 120.1	47.6 ± 136.5	82.2 ± 199.9	99.6 ± 162.6	0.039
≥ 200 mL (number, %)^c^	32 (10.2%)	3 (5%)	8 (7.8%)	9 (10.1%)	12 (19.4%)	0.044
**Complications (number, %)**^**c**^	46 (14.6%)	4 (6.7%)	12 (11.7%)	14 (15.7%)	16 (25.8%)	0.018
**Hospital stay (days)**^**a**^	5.8 ± 2.5	4.3 ± 1.4	5.2 ± 1.5	5.8 ± 1.6	8.4 ± 3.7	0.000

^a^mean ± standard deviation, tested by analysis of variance; ^b^median (interquartile range), analyzed by rank sum tests; ^c^ analyzed by Kruskal- Wallis tests; *CS* cesarean section, *CSP* cesarean scar pregnancy, *hCG* human chorionic gonadotrophin, *UAE* uterine artery embolization

Thirty-two patients (10.2%) had intraoperative blood loss ≥200 mL during curettage. Univariate analysis showed that there were significant differences in gestational age, CSP mass diameter, myometrial thickness, blood supply, type of CSP, and treatment interval (the timing of curettage after UAE) between the bleeding group and control group (Table 2). Accordingly, these variables were identified as potential risk factors for hemorrhage in this combination treatment strategy. Multivariable analysis was further conducted to adjust for confounding factors. These candidate risk factors were divided into two categories and analyzed in multivariable binary logistic regression models of intraoperative bleeding (Table 3). The cutoff of each noncategorical variable was selected depending on the median value in bleeding cases. In the final model, an interval between UAE and curettage > 72 h was identified as one of the four significant risk factors, with an adjusted OR of 3.37 (95% CI: 1.40-8.09, *P* = 0.007). The other three risk factors were myometrial thickness ≤ 0.1 cm (adjusted OR 4.55, 95% CI: 1.73-11.94, *P* = 0.002), maximum diameter of CSP mass ≥ 5 cm (adjusted OR 3.86, 95% CI: 1.53-9.74, *P* = 0.004), and type II CSP (adjusted OR 3.00, 95% CI: 1.25-7.21, *P* = 0.014). To discuss the optimal timing of curettage, we further set different cutoffs of the treatment interval in the multivariable logistic regression. When the treatment interval was divided at the cutoff of 48 h, the adjusted OR was 2.2 (95% CI: 0.94-4.92, *P* = 0.068). The variable with a cutoff at 24 h had an adjusted OR of 2.1 (95% CI: 0.59-7.77, *P* = 0.243).

**Table 2:** Univariate analysis: the candidate risk factors of intra-operative bleeding

Factors	Cases	Controls	***P*** value
	***N*** = 32	***N*** = 282	
**Maternal age (years)**^**a**^	32.3 ± 4.8	31.8 ± 5.2	0.641
**Gravidity**^**b**^	4 (2-10)	4 (1-11)	0.978
**Parity**^**b**^	1 (1-2)	1 (1-3)	0.097
**Number of prior CS**^**b**^	1 (1-2)	1 (1-3)	0.083
**Interval between CS and CSP (years)**^**a**^	6.1 ± 4.4	5.7 ± 3.9	0.224
**Recurrent CSP (number, %)**^**c**^	3 (9.4%)	8 (2.8%)	0.090
**Fetal heart beat (number, %)**^**c**^	7 (21.9%)	105 (37.2%)	0.060
β-hCG level (*10^5mIU/mL)^**a**^	7.01 ± 6.61	5.64 ± 6.96	0.109
**Gestational age (days)**^**a**^	58.4 ± 14.9	50.5 ± 10.1	0.000
**Maximum diameter of CSP mass (cm)**^**a**^	4.6 ± 2.3	3.0 ± 1.4	0.003
**Median diameter of CSP mass (cm)**^**a**^	3.7 ± 1.8	2.3 ± 1.2	0.001
**Minimum diameter of CSP mass (cm)**^**a**^	2.9 ± 1.7	1.8 ± 1.1	0.000
**Myometrial thickness (cm)**^**a**^	0.03 ± 0.06	0.12 ± 0.08	0.027
**Rich blood supply (number, %)**^**c**^	12 (37.5%)	44 (15.6%)	0.002
**Type of CSP**			
Type I (number, %)^c^	19 (53.4%)	244 (86.5%)	0.000
Type II (number, %)^c^	13 (40.6%)	38 (13.5%)	
**Interval between UAE and curettage (hours)**^**#**^	72 (24-168)	48 (12-168)	0.003

^a^ mean ± standard deviation, analyzed by independent-sample *t* tests; ^b^ median (interquartile range), analyzed by rank sum tests; ^c^ analyzed by chi-square tests; *CS* cesarean section, *CSP* cesarean scar pregnancy, *hCG* human chorionic gonadotrophin, *UAE* uterine artery embolization

**Table 3:** Adjusted odds ratios (ORs) of significant variables in the final binary logistic regression model

Risk factor	OR (95%CI)	P value	B	Wald
**Maximum diameter of CSP mass ≥ 5 cm**	3.86 (1.53-9.74)	0.004	1.35	8.15
**Type II CSP**	3.00 (1.25-7.21)	0.014	1.10	6.02
**Myometrial thickness ≤ 0.1 cm**	4.55 (1.73-11.94)	0.002	1.52	9.45
**Interval between UAE and curettage >72 h**	3.37 (1.40-8.09)	0.007	1.21	7.35
Constant −3.99				

## Discussion

One of the main challenges in the management of CSP is massive bleeding. Uterine artery embolization, which has been widely used in the field of gynecology and obstetrics to control hemorrhages, is regarded as a good option for treating CSP with minimal invasion, especially when used as an adjuvant therapy with other surgical treatments, such as curettage. There have been dozens of studies evaluating the efficacy and safety of UAE used in CSP treatment, either as a single therapy or combined with other methods [5–10, 15–20].

However, currently, no study has reported the optimal timing of surgical treatment after prophylactic UAE. The previously reported study designs were quite different in terms of the treatment interval between UAE and curettage.

During the UAE procedure, MTX was locally injected through the uterine arteries followed by the main stems of uterine arteries blocked by gelfoam particles [21]. MTX works to kill embryonic and trophoblastic cells. The reduction in uterine blood supply by UAE was impermanent, since the gelfoam could be resolved within 7-14 days. Depending on this mechanism, most authors theoretically suggested that curettage should be performed within 24-72 h after UAE to balance the benefit of onset time and the risk of recanalization [5–11]. Currently, this is only a clinical opinion, and it still needs support from reliable clinical evidence. In our hospital, gynecologists performed curettage within 24 h or longer than 72 h after UAE in some cases. The treatment intervals were individualized depending on the operation schedule, the gynecologist’s intentions, or patient preference. Thus, we designed the present study to discuss the most appropriate time to perform curettage after UAE.

In this cohort study, we found that patients in the short-interval arm were likely to have better clinical outcomes than those in the long-interval arms. This result demonstrated that the delay of curettage following UAE might increase the risk of intraoperative bleeding and other complications. Consistently, the results of multivariable logistic regression indicated that a treatment interval longer than 72 h after UAE was one of the risk factors for intraoperative bleeding. This increase in bleeding risk over time might be explained by the initiation of collateral circulation or tissue edema due to long-term ischemia. The incidence of bleeding was lower in shorter-interval arms, which indicated that a treatment interval within 24 h might be ideal. However, when we adjusted the cutoff of this variable at 24 or 48 h in the multivariable analysis, the statistical results showed no significance. Therefore, we could not form a conclusion about the best timing of curettage after UAE based on this study, but we strongly suggest that curettage not be delayed longer than 72 h under general conditions.

Other risk factors identified as risk factors for bleeding in this combination therapy included lower myometrial thickness, larger diameter of the CSP mass, and type II CSP. Since this finding coincides with our previous study [15] and other reports [22, 23], we do not discuss these factors more. However, gestational age and peritrophoblastic blood supply, which were identified as potential risk factors in the univariate analysis, were not included in the results of our final regression model. This result might be explained by (1) the synergy of gestational age and diameter of the gestational sac (adjusted as a confounding factor) and (2) the successful blockage of the blood supply by UAE, thereby removing its influence on the clinical outcome in this combination treatment strategy.

There are limitations of our study. First, this is a retrospective and observational study. The interventions were decided depending on the intentions of clinicians and patients, with selection bias. Second, the bleeding risk factors in the treatment of CSP are multifactorial and interactive. Our study showed that the influence of treatment interval had significant clinical and statistical meaning but that it might not be an independent factor. Last, even though our sample size was large enough for a study from a single medical center, multicenter randomized controlled trials (RCTs) with more reliable evidence are needed to reach a better conclusion.

## Conclusions

In the combination treatment of adjuvant UAE plus subsequent curettage for CSP, patients who have a shorter time interval between the two procedures are likely to have a lower risk of bleeding than those in the longer-interval group. A treatment interval > 72 h is a significant risk factor for bleeding during curettage. We strongly suggest that curettage not be delayed longer than 72 h after UAE under general conditions. However, more clinical trials, such as RCTs, are necessary to form a final conclusion on when to perform curettage after UAE.

## Data Availability

All data generated or analysed during this study are included in this published article.

## Abbreviations

UAE: Uterine artery embolization
CSP: Cesarean scar pregnancy
CS: Cesarean section
MTX: Methotrexate
beta-hCG: Beta-human chorionic gonadotrophin
MRI: Magnetic resonance imaging
SD: Standard deviation
IQR: Interquartile range
ANOVA: Analysis of variance
OR: Odds radio
CI: Confidence interval
RCTs: Randomized controlled trials
